# Environmental Enrichment Sharpens Sensory Acuity by Enhancing Information Coding in Barrel Cortex and Premotor Cortex

**DOI:** 10.1523/ENEURO.0309-20.2021

**Published:** 2021-05-14

**Authors:** He J. V. Zheng, Jesse P. Meagher, Duo Xu, Yogi A. Patel, Daniel H. O’Connor, Hyung-Bae Kwon

**Affiliations:** 1 Max Planck Florida Institute for Neuroscience, Jupiter, FL 33458; 2 Department of Neuroscience, Johns Hopkins University School of Medicine, Baltimore, MD 21205; 3 Department of Biomedical Engineering, Georgia Institute of Technology, Atlanta, GA 30332; 4 Max Planck Institute of Neurobiology, Martinsried 82152, Germany

**Keywords:** enriched environment, premotor cortex, somatosensory cortex, tuning heterogeneity, two-photon imaging, whisker discrimination task

## Abstract

Environmental enrichment (EE) is beneficial to sensory functions. Thus, elucidating the neural mechanism underlying improvement of sensory stimulus discrimination is important for developing therapeutic strategies. We aim to advance the understanding of such neural mechanism. We found that tactile enrichment improved tactile stimulus feature discrimination. The neural correlate of such improvement was revealed by analyzing single-cell information coding in both the primary somatosensory cortex and the premotor cortex of awake behaving animals. Our results show that EE enhances the decision-information coding capacity of cells that are tuned to adjacent whiskers, and of premotor cortical cells.

## Significance Statement

This study advances the understanding of the neural mechanisms underlying the improvement of tactile discrimination induced by tactile environmental enrichment (EE). We demonstrate that enrichment improves the information-coding capacity of adjacent-whisker tuned cells in the barrel cortex and premotor cortex, in awake animals performing a single whisker discrimination task. This understanding contributes to the development of therapeutic strategies for sensory function improvement using EE, which is a promising non-invasive therapy for many neurodegenerative diseases and traumatic brain injury and stroke recovery.

## Introduction

Neural plasticity, the basis for learning, memory, and development, is heavily dependent on sensory experience, even in adulthood (Wiesel and Hubel, 1963; Diamond et al., 1993; Zito and Svoboda, 2002; Karmarkar and Dan, 2006; Holtmaat and Svoboda, 2009; Fu and Zuo, 2011). Positive sensory experience, most prominently environmental enrichment (EE; or enrichment), where novel objects, complex surroundings, and/or social interaction provide sensory and cognitive stimulation and encourage physical and exploratory activity, is known to enhance neural plasticity (Diamond et al., 1972, 1976; Greenough and Volkmar, 1973; Greenough et al., 1973, 1985; Connor et al., 1982; Turner and Greenough, 1985; Rampon et al., 2000; Faherty et al., 2003). EE also improves cognitive behavior (Van Praag, 2000; Leggio et al., 2005), ameliorates neurodegenerative disease symptoms (van Dellen et al., 2000; Hockly et al., 2002; Arendash et al., 2004; Jankowsky et al., 2005; Nithianantharajah and Hannan, 2006), and aids recovery from traumatic brain injuries and stroke (Hicks et al., 2007; Janssen et al., 2010; Kovesdi et al., 2011; Alwis and Rajan, 2014). Enrichment has also been shown to improve sensory function and stimulus discrimination. For example, enriched housing improves the visual function of amblyopic rats (Sale et al., 2007; Baroncelli et al., 2012; Tognini et al., 2012). Olfactory enrichment results in an improved ability to discriminate between odor pairs (Mandairon et al., 2006a,b). Similarly, EE enhances spatial discrimination of sound source, with faster reaction times and improved discrimination accuracy (Cai et al., 2009). Given the ethological relevance and therapeutic potential, it is important to fully elucidate the neural mechanisms of how EE improves the animal’s ability to discern features of external stimuli. However, how sensory information is encoded under EE at single cell level in primary sensory cortex and frontal areas that are related to perceptual decision-making, especially in the awake brain, has not been shown.

We used the rodent vibrissal pathway as a model to investigate the cellular mechanism of how EE may improve stimulus feature discrimination, because it is an excellent model for both sensory processing and experience-dependent plasticity. Neurons within a column in vibrissal somatosensory cortex (vS1) respond most vigorously to a single whisker (principal whisker) stimulation, and the topography of the columns match the whisker arrangement (Woolsey and Van der Loos, 1970; Simons, 1978), forming a “barrel map.” Furthermore, the adult barrel cortex, especially layer 2/3 (L2/3), exhibits experience-dependent plasticity (for review see Feldman and Brecht, 2005). Dramatic changes in the spatial representation of a single whisker occur under conditions including learning whisker-based discrimination tasks, being housed in a naturalistic environment or EE (Polley et al., 2004; Guic et al., 2008; Devonshire et al., 2010), overstimulation of a whisker (Mégevand et al., 2009), and sensory-deprivation by whisker trimming (Diamond et al., 1993). However, the effect of EE on the spatial representation of a single whisker is unclear. Some studies demonstrated diminished spatial spread (Polley et al., 2004; LeMessurier et al., 2019), while others reported broadened spatial spread (Guic et al., 2008; Devonshire et al., 2010). Hence, it is not clear whether EE may improve whisker sensory acuity by sharpening the spatial representation or by strengthening the response to single-whisker stimulation in adjacent columns. Two reasons suggest that the latter is the more likely scenario. First, L2/3 cells in primary sensory cortices have long been speculated to integrate input from adjacent columns (Brumberg et al., 1996; Moore and Nelson, 1998; Chelaru and Dragoi, 2008; Adesnik and Scanziani, 2010; Wester and Contreras, 2012; Hawkins et al., 2017). Second, EE encourages the formation of new synapses in the barrel cortex (Greenough and Volkmar, 1973; Greenough et al., 1973, 1985; Connor et al., 1982; Turner and Greenough, 1985; Landers et al., 2011), and potentiates the neural response to a single whisker stimulation (Alwis and Rajan, 2013). This led us to hypothesize that EE evokes functional plasticity in L2/3 cells by enhancing their response to an adjacent whisker stimulation, resulting in better comparison of adjacent whisker stimuli. Subsequently, vS1 may enhance its output to downstream frontal cortical areas that are related to decision-making, leading to improved decision-information coding, thus sharpening the animal’s spatial acuity when sensing whisker stimuli.

To test our hypothesis, we housed a group of mice each individually in a tactile-enriched rat cage with novel objects, toys, and tunnels of various shapes and textures that were changed at least twice a week. We designed an adjacent-whisker spatial discrimination task where mice were trained to lick for a water reward on detecting the stimulation of a designated “go” whisker, and withhold licking if an adjacent “no-go” whisker was stimulated. This task allowed us to investigate the relationship between the animal’s spatial acuity in whisker sensing and the single whisker spatial representation change in vS1 induced by EE. In haptic perception, previous studies show that EE has no effect on the animal’s ability to discriminate textured surfaces (Finger and Fox, 1971; Bourgeon et al., 2004). It is possible that textured surface discrimination is not difficult enough of a task to reflect the effect of EE. Therefore, it is important to probe the most basic sensor spatial acuity with an adjacent-whisker discrimination task.

Both enriched and standard-housed control mice performed the adjacent whisker discrimination task. Simultaneously, we used two-photon calcium imaging to record single-cell activity in vS1 or vibrissal premotor cortex (vM2). In rodents, M2 forms reciprocal connections with S1 (Reep et al., 1984, 1987, 1990) and serves important roles in sensory processing and decision-making (Vargo et al., 1988; Sul et al., 2011; Murakami et al., 2014; Manita et al., 2015). Therefore, we chose vM2 as a decision-making area to investigate whether choice-related information was improved in enriched animals.

## Materials and Methods

### Animals

Wild-type mice C57BL/6 [postnatal day (P)30–P40] were used for these experiments (Charles River Laboratory). All mice were on a reverse light cycle, and housed individually in their own cage. For vS1 imaging, six mice (three male, three female) were enriched, and five mice (three male, two female) were in the control group. For vM2 imaging, seven mice (three male, four female) were enriched, and four mice (two male, two female) were in the control group. Behavior data were collected from seven enriched mice (four male, three female) and six control mice (three male, three female). Mice used for imaging groups (both vS1 group and vM2 group) were used for behavior analysis. All experiments were in compliance with the Institutional Animal Care and Use Committee and National Institutes of Health guidelines.

### Surgery

Mice were anesthetized with a ketamine/xylazine cocktail (8.0 mg ketamine + 0.6 mg xylazine/ml, 10 ml/kg, i.p.). Standard aseptic sterile surgery techniques were applied for survival surgeries. The animal was kept on a thermal pad to maintain a body temperature of 37°C. The animal was head-fixed in a stereotaxic frame with non-penetrating ear bars. Before the initial incision, the hair was removed, and the scalp was cleaned with 70% ethanol and 10% povidone-iodine solution. The scalp was removed. The injection coordinates over vS1 (1.6 mm posterior, 3.3 mm lateral) and/or vM2 (1.5 mm anterior, 0.5 mm lateral) relative to bregma were measured. A burr hole was drilled at the injection sites. An injection pipette (inner diameter ∼260 μm, tip diameter ∼15 μm, BRAND) was then filled with 400 nl of AAV1-Syn-GCamp6s-WPRE-SV40 solution (Addgene). The pipette was slowly inserted into the burr hole ∼300 μm below pia. The virus solution was infused by a micropump at a flow rate of 0.9 μl/min. A craniotomy 3 mm in diameter was drilled over the virus injection site. The cortex was cleaned and covered with a 3 mm round glass coverslip (Warner Instruments). Dental cement (C&B Metabond, Parkell) was then used to seal the coverslip to the skull and to cement a head plate onto the skull.

### Behavior training

#### 

##### Acclimation

The animals were allowed at least 5 d of recovery in a reversed light cycle room after surgery, then water restricted (1 ml/d) for 7–10 d. The animals were handled and watered in the imaging rig to acclimate to the experiment setup, then trained to tolerate head fixation. The head fixation duration starts at 5 min at a time for the first day, and gradually increased until mice were calmly head fixed for 1 h.

#### Enrichment

**Figure 1.**
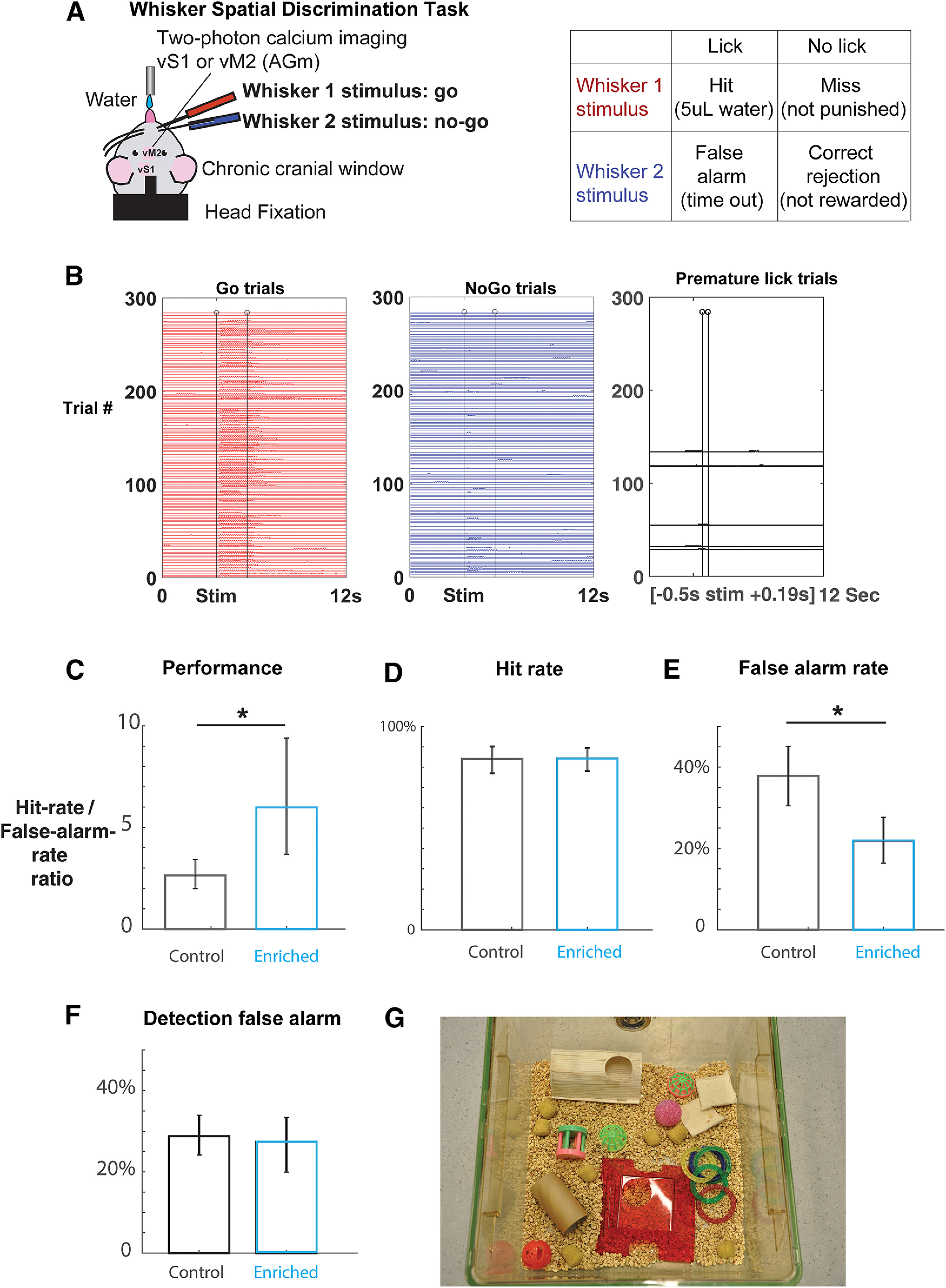
Tactile environment enrichment sharpens spatial acuity in adjacent-whisker discrimination. ***A***, Awake, head-fixed mice are trained on a whisker spatial discrimination go/no-go task while being simultaneously imaged. The animal licks for water reward on the same go whisker stimulation, but withholds licking on any other whisker stimulation. In the final form of the task, the no-go whisker is always adjacent to the go whisker. ***B***, An example lick raster of a discrimination task session. Go trials (left) and no-go trials (middle) are randomly interleaved. Each trial is 12 s, and the stimulus onset is at 3.5 s (first vertical line). The animal has a response window of 0.2–2 s after stimulus onset (second vertical line signifies the end of response window). Trials where animals lick prematurely (0.5 s before and 0.19 s after stimulus onset) are excluded. A well-trained animal typically has <5% premature trials. Tactile task performance is measured by the ratio of hit rate to false alarm rate. In this example session, the animal’s discrimination performance is 4.67. ***C***, Discrimination task performance is significantly better in enriched animals (control bootstrap mean = 2.64, error bar: 2.5th percentile = 1.99, and 97.5th percentile = 3.43, *N* = 7885 trials, 6 mice; enriched bootstrap mean = 5.98, error bar: 2.5th percentile = 3.68, and 97.5th percentile = 9.40, *N* = 13,123 trials, 7 mice; *p* < 0.05). ***D***, The hit rates were the same between control and enriched animals (control bootstrap mean = 84.07%, error bar: 2.5th percentile = 76.92%, and 97.5th percentile = 90.17%, *N* = 7885 trials, 6 mice; enriched bootstrap mean = 84.36% error bar: 2.5th percentile = 78.09%, and 97.5th percentile = 89.52%, *N* = 13,123 trials, 7 mice; *p* > 0.05). ***E***, Lowered false alarm rate is the main behavior improvement in enriched animals (control bootstrap mean = 37.82%, error bar: 2.5th percentile = 30.49%, and 97.5th percentile = 45.17%, *N* = 7885 trials, 6 mice; enriched bootstrap mean = 21.86% error bar: 2.5th percentile = 16.37%, and 97.5th percentile = 27.64%, *N* = 13,123 trials, 7 mice; *p* < 0.05). ***F***, The detection false alarm rates were not different between enriched and control animals (control bootstrap mean = 28.79%, error bar: 2.5th percentile = 24.15% and 97.5th percentile = 33.92%, *N* = 7829 trials, 6 mice; enriched bootstrap mean = 27.39%, 2.5th percentile = 19.93% and 97.5th percentile = 33.44%, *N* = 15 073 trials, 7 mice; *p* > 0.05). ***G***, An example of a large cage with objects of various shapes and textures used for tactile enrichment. *p < 0.05.

After the animals could tolerate head fixation long enough (∼30 min) for a baseline imaging session to obtain a barrel map (typically after 7–10 d of acclimation and training), approximately half were randomly assigned to the enriched group. Each mouse in enriched groups was placed in a large rat cage (∼43 cm in length × 30 cm in width × 20 cm in height) individually, with objects and toys of various shapes and texture for ∼8 h during the day. An example of such a cage is shown in Figure 1*G*. Objects were changed and/or rearranged twice a week. For nights and weekends, to be still housed on a mouse rack, the enriched mice were housed individually in a mouse cage (∼30 cm in length × 22 cm in width × 14 cm in height) with a reduced amount of toys and objects. Each mouse in control groups was housed individually in a mouse cage, with a layer of bedding material and a food bowl. Enrichment continued as the animals learned and performed the task (typically four to six weeks).

#### Detection task

Mice were first trained on a detection task, before they can progress to the discrimination task. The animals were first trained to associate the mechanical stimulation of a designated go whisker with automatic water reward. Each trial was 12 s long. A sound cue (2500 ± 300 Hz, 0.5 s) signaled the beginning of the trial. The whisker was deflected in the rostral-caudal direction (10 Hz, ∼676°/s, 0.5 s) at 3.543 s after trial onset. At association stage, ∼5 μl of water was automatically delivered 0.2 s after the onset of whisker stimulation. Once the mice learned to consistently lick for water following the whisker stimulation, they were given a response window (0.2–2 s after stimulus onset) to lick the water spout to trigger a water reward instead of receiving automatic water reward. Initially all trials are go trials where a whisker stimulation was presented. Once they reached a hit rate of ∼80%, trials with no whisker stimulus (no-go trials) were randomly and gradually mixed in until they consisted of 50% of the trials. During the response window in a go trial, if the mouse licked for water, it was classified as a “hit,” otherwise a “miss.” Hits were rewarded with ∼5 μl of water, and misses were not punished. Mice were trained to withhold licking during no-go trials. If the animal licked for water during the response window, it was a “false alarm,” otherwise a “correct rejection” (Fig. 1*A*). False alarms were punished with delays to the next trials until the animal withhold licking. Correct rejections were not rewarded. Once an animal’s performance (hit-rate to false-alarm-rate ratio) reached 2, it was considered that they had learned the task.

#### Discrimination task

After the mice learned detection, they were trained on the discrimination task. The no-go trials now presented the stimulation of a no-go whisker (a whisker that was not the designated go whisker). No-go trials were again randomly and gradually mixed in until they consisted of 50% of the trials. The no-go whisker started at a location remote from the go whisker, then was gradually moved toward the go whisker until they were adjacent. Each animal typically tolerated head-fixation for ∼1 h at a time (one single session), and performed ∼200–300 trials during this hour and became sated with water by the end of the hour/session.

### Whisker stimulation

Whisker stimulation was controlled by an analog galvanometer motor (Cambridge Technology) with a custom attachment to allow a single whisker to be threaded into a small capillary (inner diameter 0.50 mm). The end of the capillary was situated ∼2–3 mm from the face. When two adjacent whiskers were stimulated, care was taken so that the whisker capillaries did not touch each other when in motion. A Gaussian white noise was used to mask any sound from the motors. All devices during the behavior training were controlled and the data recorded using a real-time program custom written in the Real-Time eXperiment Interface (RTXI) framework (Patel et al., 2017).

### *In vivo* functional imaging

Wide-field epifluorescence imaging was used to identify the area of activation in response to whisker stimulation. The mouse was head-fixed. An objective lens (either 4× magnification, 0.10 NA, 18.5 mm WD, Olympus, or 16×, 0.80 NA, 3.0 mm WD, Nikon) was placed over the cranial window and the cortical surface was illuminated with a blue LED (470 nm, Thorlabs). Fluorescence signal was collected with a CCD camera (QIMAGING) at 168 ms per frame and 310 pixels/mm (4×) or 1120 pixels/mm (16×). The field of view was ∼2.24 × 1.67 mm for 4× and 621 × 464 μm for 16×.

Two-photon calcium imaging was used to record individual cell activity in L2/3. A 16× lens was placed over the cranial window and water was filled in between. A 920 nm laser (Spectra-Physics) was used to excite the GCamp (∼15 mW) and the fluorescence signal was collected with a photomultiplier tube (H7422A-40, Hamamatsu) at 1.18 s per frame at 786 pixels/mm (Prairie View, Bruker Imaging). The field of view was ∼651 × 651 μm.

### Histology

Mice were perfused with 4% paraformaldehyde. The cortex was peeled, flattened to 1 mm and sliced tangentially at 100 μm on a Vibratome (VT1200S, Leica). The barrel field was visualized under a bright field microscope with a 4× objective lens.

### Quantification and statistical analysis

All raw images were first registered for micromotion in ImageJ (TurboReg Plugin). Cells were manually selected using standard deviations projection (Cell Magic Wand Plugin), and the centroid and mean gray value of each cell over time (ΔF) were calculated and exported to MATLAB for analysis. For each cell, a change in fluorescence relative to the background (ΔF/F_0_) was calculated, where F_0_ is the average of the first two frames. Error bars in all figures indicate 1 SE.

#### Whisker tuning

**Figure 2.**
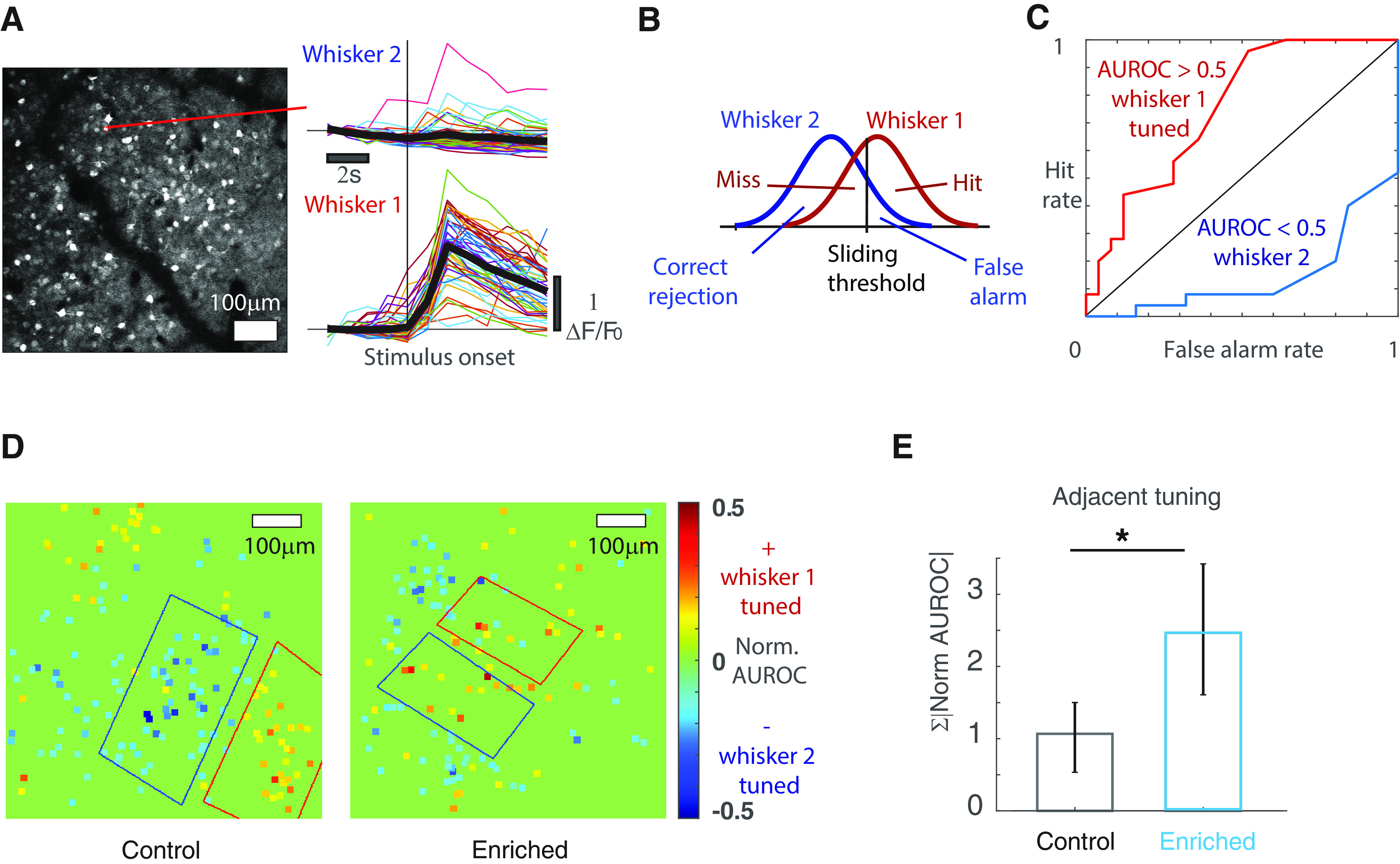
EE increases whisker adjacent-tuning in vS1. ***A***, An example two-photon calcium image of vS1 L2/3 response to a single-whisker stimulation in an awake, head-fixed mouse. Right, The response of an example cell (outlined in red) to two adjacent whisker stimulation in separate, interleaved trials. Thin colored lines are single trials of calcium trace, thick black lines are trial-averaged. ***B***, Whisker tuning calculation: the maximum response from all trials of whisker 1 stimulation form a distribution while those from whisker 2 stimulation trials form the other. ROC analysis is used to quantify the separation of the two distributions. ***C***, Two example ROC curves from two individual cells. An AUROC above 0.5 signifies tuning to whisker 1, and below 0.5 to whisker 2. ***D***, Examples of L2/3 cell whisker tuning in vS1 (left: vS1 of a control mouse, right: vS1 of an enriched mouse). The AUROC is normalized by subtracting 0.5, so that positive values signify tuning to whisker 1, and negative values to whisker 2. Warm-colored dots: cells tuned to whisker 1 at its physical location; cold-colored dots: cells tuned to an adjacent whisker 2. Red outline: approximate functional space of whisker 1 principal barrel, identified using wide-field imaging. Blue outline: approximate functional space of whisker 2 principal barrel. ***E***, Adjacent whisker tuning increases in enriched animals. Adjacent tuning is defined as the total absolute value of normalized AUROC of all the cells tuned to the adjacent whisker within the approximate functional boundary of a given barrel (control bootstrap mean 1.07, error bar: 2.5th percentile 0.53, and 97.5th percentile 1.50, *N* = 60 AT cells, 5 mice; enriched bootstrap mean 2.47, error bar: 2.5th percentile 1.61, and 97.5th percentile 3.42, *N* = 108 AT cells, 6 mice; *p* < 0.05). *p < 0.05.

For each single cell, the maximum response up to two frames after stimulus onset was collected from each trial. Values from go whisker stimulation trials form a distribution while those from no-go whisker stimulation trials form another (Fig. 2*B*). The area under the receiver operating characteristic curve (AUROC) of the two distributions was calculated and normalized by subtracting 0.5, so that a positive normalized AUROC signifies go whisker preference, and negative value signifies no-go whisker preference (Fig. 2*C*). If a cell’s principal barrel and preferred whisker were different, it was classified as an adjacent-tuned (AT) cell, otherwise a principal-tuned (PT) cell. To determine a cell’s principal barrel, the centroids of go and no-go barrels were calculated from a 10-trial average 16× wide-field image of cortical activation in response to a given whisker stimulation and transformed onto the two-photon imaging background. The *x*- and *y*-plane coordinate translation from 16× wide-field image to two-photon image was previously calibrated. An anatomic barrel map (as we found no significant deviations among individual animals) was then scaled and rotated, so that the centroids of go and no-go barrels and the functional centroids coincide.

#### Behavior analysis

The performance of an animal was calculated by the ratio of hit rate to false alarm rate. A ratio of 2 signifies that the animal is trained on the task. Any trial where the animal licked prematurely in the time window [−0.19 0.2 s) relative to stimulus onset was eliminated from analysis. A trained animal typically has <5% such trials (Fig. 1*B*).

#### Decoding analysis

Fisher linear discriminant analysis (LDA) was used to predict the animal’s behavior given single-trial cellular population response. When using different classes of cells (e.g., AT cells vs PT cells), the cell numbers were kept the same for each class. From each performance category (false alarm vs correct rejection), half of the trials were randomly chosen to train the classifier, and the other half were used to test the classifier accuracy. The decoder performance is the % of trials correctly predicted in the test trials. If a particular performance category has <50 trials for either training or test data, then it is randomly resampled. This process is iterated 10 times and an average over 10 iterations is the recorded performance.

#### Hierarchical multilevel bootstrap analysis

**Figure 3.**
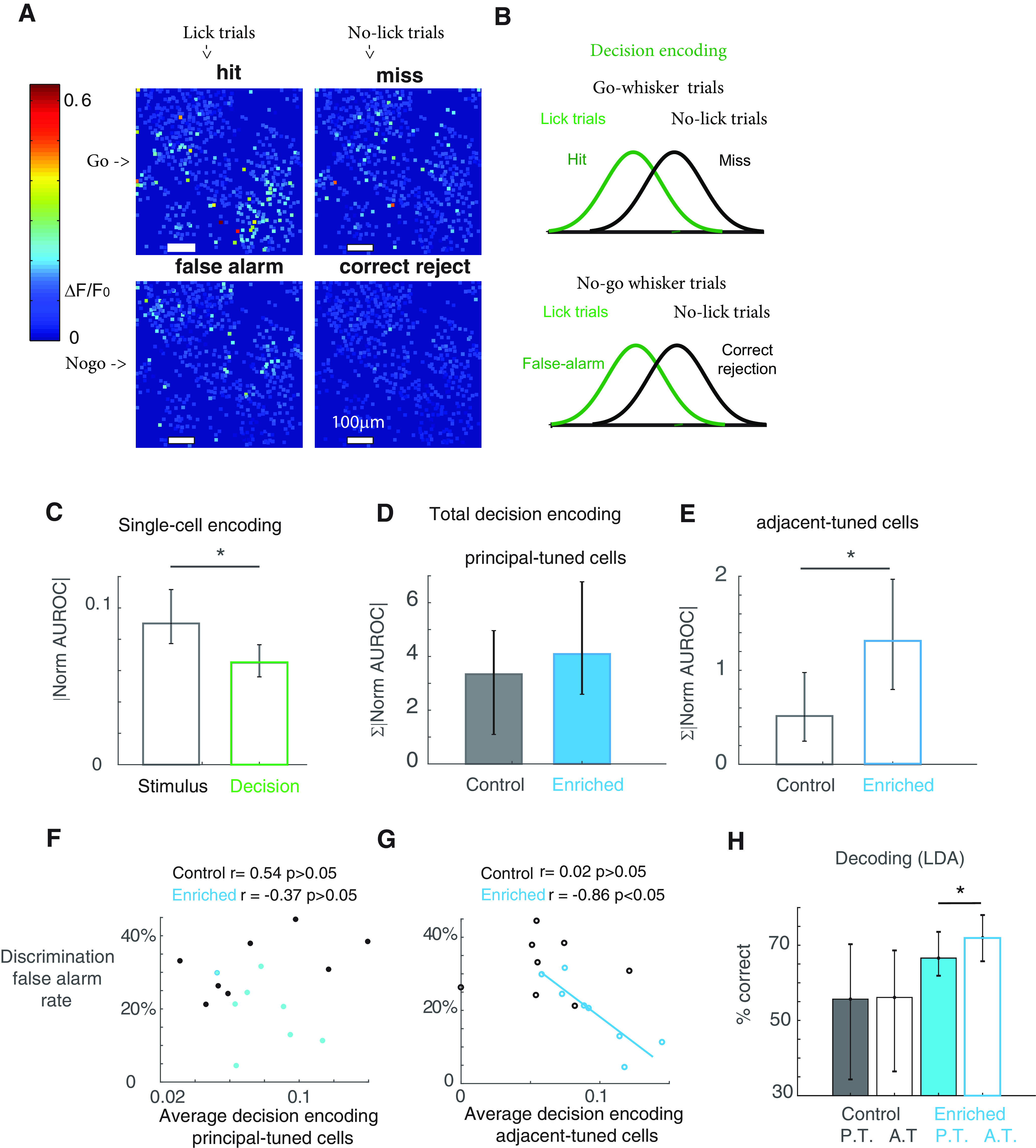
Function of adjacent-tuned cells explains behavior in enriched animals. ***A***, An example session of S1 L2/3 cellular activity in an awake animal performing a whisker discrimination task. Images are averaged over all trials within a performance criterion. ***B***, For a single cell, decision encoding is quantified by ROC analysis between the response distributions during the trials where the animal licked versus those where the animal did not lick. It is calculated under conditions where go whisker was stimulated versus no-go whisker was stimulated. ***C***, S1 cells encode stimulus feature more than decision. The information about the stimulus and decision is averaged (mean of absolute value of normalized AUROC over all cells). Because the determining factor in discrimination performance is the false alarm rate, all decision coding in this study refers to the decision between false alarm and correct rejection trials (bottom of ***B***). Stimulus encoding bootstrap mean = 0.090, error bar: 2.5th percentile = 0.077, and 97.5th percentile = 0.11; decision encoding bootstrap mean = 0.065, error bar: 2.5th percentile = 0.056, and 97.5th percentile = 0.077, *N* = 7092 cells, 11 mice; *p* < 0.05. ***D***, In PT cells, enrichment does not improve their decision-coding capacity (control bootstrap mean 3.34, error bar: 2.5th percentile 1.10, and 97.5th percentile 4.96, *N* = 361 PT cells, 5 mice; enriched bootstrap mean 4.09, error bar: 2.5th percentile 2.59, and 97.5th percentile 6.78, *N* = 400 PT cells, 6 mice; *p* > 0.05). ***E***, AT cells encode more decision information with enrichment (control bootstrap mean 0.51, error bar: 2.5th percentile 0.25, and 97.5th percentile 0.97, *N* = 60 AT cells, 5 mice; enriched bootstrap mean 1.31, error bar: 2.5th percentile 0.80, and 97.5th percentile 1.97, *N* = 108 AT cells, 6 mice; *p* < 0.05). ***F***, Decision information encoded in PT cells cannot predict behavior (control animals *r* = 0.54, *p* > 0.05, bootstrap portion of samples that have significant correlation was 23.2%, the mean of significant correlation value is 0.62, *N* = 361 PT cells, 5 mice; enriched animals *r* = −0.37, *p* > 0.05, bootstrap portion of samples that have significant correlation was 24.3%, the mean of significant correlation value is −0.75, *N* = 400 PT cells, 6 mice). ***G***, In enriched animals, but not control animals, the average decision information encoded in AT cells predicts false alarm rate in discrimination task (enriched: *r* = −0.86, *p* < 0.05, in multilevel hierarchical bootstrapped samples, portion of samples that have significant correlation was 55%, the mean of significant correlation value is −0.82, *N* = 108 AT cells, 6 mice; control: *r* = 0.02, *p* > 0.05, bootstrap portion of samples that have significant correlation was 7.4%, the mean of significant correlation value is −0.28, *N* = 60 AT cells, 5 mice). ***H***, Using populations of either PT cells or AT cells, Fisher LDA is used to classify false alarm and correct rejection trials. For any given session, the number of PT cells and the number of AT cells are kept the same. The decoder predicts a single trial more accurately using AT cells than PT cells in enriched animals, but not in control animals (control PT bootstrap mean 56%, error bar: 2.5th percentile 35%, and 97.5th percentile 71%, AT bootstrap mean 56%, error bar: 2.5th percentile 36% and 97.5th percentile 68%, *N* = 60 cells, 5 mice; enriched PT bootstrap mean 67%, error bar: 2.5th percentile 62%, and 97.5th percentile 74%, AT bootstrap mean 72%, error bar: 2.5th percentile 66% and 97.5th percentile 78%, *N* = 108 cells, 6 mice). *p < 0.05.

We used hierarchical multilevel bootstrap analysis (Davison and Hinkley, 1997; Kwon et al., 2016; Saravanan et al., 2020; Xu et al., 2020) to resolve the issues of dependency in the data and relatively small sample sizes. For each statistic, at each level of data collection (animal, session, then cell and/or trial), we randomly resample with replacement and recalculate the statistic. As an example, we describe here the process of bootstrapping adjacent tuning (Fig. 2*E*) for the control group. First, we plot adjacent tuning from the eight sessions out of the five animals, and excluded any outliers using the box plot. Then, we randomly resampled the five animals with replacement. For each resampled animal, we randomly resampled the sessions recorded. Finally, for each resampled session, we resampled the cells with replacement to calculate the adjacent tuning. This process is iterated 1000 times. Each iteration yields a new statistic per session, and the mean of these statistics was recorded for that single iteration. Finally, 1000 bootstrapped statistics for the control group were tested against those of the enriched group. To perform statistical significance testing, we first calculated randomized differences of means. For each bootstrap iteration, we shuffled the bootstrapped adjacent tuning from both control and enriched groups, then randomly reassigned them into two groups. The difference between the mean of the two random groups was recorded for that single iteration. The 97.5th percentile value of the randomized differences signifies the 5% probability (*p* = 0.05) that, the observed difference of mean (mean of enriched group minus mean of control group) is not larger than that if the two groups came from the same condition. For paired comparison (one-sample test, e.g., Fig. 3*H*, decoding using AT cells vs PT cells in the same animal), the difference between the pair of bootstrapped samples was calculated. If 97.5% of the difference values is >0, then we conclude the difference in sample mean is >0.

## Results

### Tactile environment enrichment sharpens spatial acuity in adjacent-whisker discrimination

Although EE proves beneficial to sensory stimulus discrimination (Mandairon et al., 2006a,b; Sale et al., 2007; Cai et al., 2009; Baroncelli et al., 2012; Tognini et al., 2012), previous studies show no improvement in surface texture discrimination (Finger and Fox, 1971; Bourgeon et al., 2004). This is possibly because textured surface discrimination is not difficult enough of a task to reflect the effect of EE. Therefore, we examined tactile discrimination using an adjacent-whisker spatial discrimination task. This task allows us to probe the basic sensor spatial acuity. To test whether enrichment could improve this spatial acuity in whisker sensing, we placed a group of mice in tactile enriched housing. Enriched mice were housed individually in a large cage with objects of various shapes and texture (for an example of such setup, see Fig. 1*G*), for ∼40 h per week. Control animals are housed individually in a small mouse cage with only bedding material and food (see Materials and Methods).

We trained head-fixed, water-restricted mice to perform a two-adjacent whisker discrimination task. The mouse was trained to lick for a water reward if a designated go whisker was deflected (Fig. 1*A*). If the mouse licked within the response window (0.2–2 s after stimulus onset), the trial was classified as a hit, otherwise a miss. In randomly interleaved no-go trials where an adjacent no-go whisker was deflected, the mouse was trained to withhold licking. If the mouse licked within the response window, the trial was a false alarm where the mouse was punished with a recursive timeout until licking stopped; otherwise, a correct rejection. Task performance was measured by the ratio of hit rate to false alarm rate. Behavior data were used for comparison between control and enriched groups after each animal had learned the task (after their performance ratio had reached 2 consistently).

Enriched animals spatially discriminated two adjacent whisker better (control bootstrap mean = 2.64, error bar: 2.5th percentile = 1.99, and 97.5th percentile = 3.43, *N* = 7885 trials, 6 mice; enriched bootstrap mean = 5.98, error bar: 2.5th percentile = 3.68, and 97.5th percentile = 9.40, *N* = 13,123 trials, 7 mice; *p* < 0.05; Fig. 1*C*). Furthermore, the driving force behind better discrimination performance was the lowered false alarm rate in enriched animals (control bootstrap mean = 37.82%, error bar: 2.5th percentile = 30.49%, and 97.5th percentile = 45.17%, *N* = 7885 trials, 6 mice; enriched bootstrap mean = 21.86% error bar: 2.5th percentile = 16.37%, and 97.5th percentile = 27.64%, *N* = 13,123 trials, 7 mice; *p* < 0.05; Fig. 1*E*), as the hit rates were not different (control bootstrap mean = 84.07%, error bar: 2.5th percentile = 76.92%, and 97.5th percentile = 90.17%, *N* = 7885 trials, 6 mice; enriched bootstrap mean = 84.36% error bar: 2.5th percentile = 78.09%, and 97.5th percentile = 89.52%, *N* = 13,123 trials, 7 mice; *p* > 0.05; Fig. 1*D*). Importantly, the false alarm rate improvement is specific to the discrimination task. In the detection task, the animals were trained to withhold licking in the no-go trials, where no whisker stimulus was presented (see Materials and Methods). The detection false alarm rates were not different between enriched and control animals (control bootstrap mean = 28.79%, error bar: 2.5th percentile = 24.15% and 97.5th percentile = 33.92%, *N* = 7829 trials, 6 mice; enriched bootstrap mean = 27.39%, 2.5th percentile = 19.93% and 97.5th percentile = 33.44%, *N* = 15 073 trials, 7 mice; *p* > 0.05; Fig. 1*F*).

### Tactile environment enrichment increases adjacent-whisker tuning in vS1

We investigated the neural correlates of the sensory acuity shown in the enriched animals. It has been reported that enrichment shrinks or broadens the spatial span of a single whisker in L2/3 (Polley et al., 2004; LeMessurier et al., 2019; Guic et al., 2008; Devonshire et al., 2010). Because enrichment encourages the formation of new synapses in the barrel cortex (Greenough and Volkmar, 1973; Greenough et al., 1973, 1985; Connor et al., 1982; Turner and Greenough, 1985; Landers et al., 2011) and potentiates the neural response to a single whisker stimulation (Alwis and Rajan, 2013), we hypothesize that EE enhances L2/3 cellular response to an adjacent whisker stimulation. This enhanced response would enable cells to better compare adjacent whisker stimuli, serving as a potential mechanism for sharpened spatial discrimination observed in behavior.

To test whether EE increased the adjacent-whisker tuning in L2/3 of vS1, we used two-photon calcium imaging to record single cell response to individual stimulation of two adjacent whiskers in awake, head-fixed mice. We then calculated the whisker preference of each cell using ROC analysis (Fig. 2*A–C*; see Materials and Methods). Figure 2*D* shows examples of cell tuning in the barrel cortex of a control (Fig. 2*D*, left) and an enriched (Fig. 2*D*, right) animal. For example, in the barrel outlined in red, cold colored cells are preferentially tuned to the adjacent whisker. We then derived a measure of adjacent tuning by summing the normalized AUROC values of all AT cells within the two barrels. After four weeks of enrichment, adjacent tuning increased in vS1 (control bootstrap mean 1.07, error bar: 2.5th percentile 0.53, and 97.5th percentile 1.50, *N* = 60 AT cells, 5 mice; enriched bootstrap mean 2.47, error bar: 2.5th percentile 1.61, and 97.5th percentile 3.42, *N* = 108 AT cells, 6 mice; *p* < 0.05; Fig. 2*E*). This result suggests that L2/3 vS1 neurons collectively increase their response to an adjacent whisker stimulation, potentially integrating more information from adjacent columns.

### AT cells carry more decision information than PT cells

L2/3 cells in primary sensory cortices have long been speculated to integrate information from adjacent columns (Brumberg et al., 1996; Moore and Nelson, 1998; Chelaru and Dragoi, 2008; Adesnik and Scanziani, 2010; Wester and Contreras, 2012; Hawkins et al., 2017). Therefore, we particularly investigated the functional change in AT cells under enrichment, especially regarding information-coding. We imaged single cell activity in L2/3 of vS1 while the animal was performing a whisker discrimination task. Figure 3*A* shows an example trial-averaged cellular activity in each performance criterion during a discrimination task session. We first analyzed how much information about the animal’s decision was encoded in vS1 cells. Because the difference in discrimination task performance was the false alarm rate, we collected the single trial responses from false-alarm trials and correct rejection trials respectively (Fig. 3*B*). We then used ROC analysis to quantify how each cell’s activity correlated with the animal’s decision during no-go trials. We found that, vS1 cells in general encoded more stimulus information than decision information. The whisker stimulus information (calculation as shown in Fig. 2) averaged over all vS1 cells was higher than the decision information (stimulus encoding bootstrap mean = 0.090, error bar: 2.5th percentile = 0.077, and 97.5th percentile = 0.11; decision encoding bootstrap mean = 0.065, error bar: 2.5th percentile = 0.056, and 97.5th percentile = 0.077, *N* = 7092 cells, 11 mice; *p* < 0.05; Fig. 3*C*).

To investigate whether AT cells underlie improved discrimination performance, we analyzed decision information encoded in AT cells and in PT cells respectively. In enriched animals, AT cells encoded more decision information (total amount of decision information summed over all AT cells) than their control counterparts (control bootstrap mean 0.51, error bar: 2.5th percentile 0.25, and 97.5th percentile 0.97, *N* = 60 AT cells, 5 mice; enriched bootstrap mean 1.31, error bar: 2.5th percentile 0.80, and 97.5th percentile 1.97, *N* = 108 AT cells, 6 mice; *p* < 0.05; Fig. 3*E*). However, PT cells showed no improvement in decision coding under EE (control bootstrap mean 3.34, error bar: 2.5th percentile 1.10, and 97.5th percentile 4.96, *N* = 361 PT cells, 5 mice; enriched bootstrap mean 4.09, error bar: 2.5th percentile 2.59, and 97.5th percentile 6.78, *N* = 400 PT cells, 6 mice; *p* > 0.05; Fig. 3*D*). Importantly, the cell-averaged decision information was well correlated with the animal’s false alarm rate in discrimination task, but only in AT cells of enriched animals (*r* = −0.86, *p* < 0.05, in multilevel hierarchical bootstrapped samples, portion of samples that have significant correlation was 55%, the mean of significant correlation value is −0.82, *N* = 108 AT cells, 6 mice; Fig. 3*G*). Such correlation does not hold in control animals (*r* = 0.02, *p* > 0.05, bootstrap portion of samples that have significant correlation was 7.4%, the mean of significant correlation value is −0.28, *N* = 60 AT cells, 5 mice) or PT cells (control animals *r* = 0.54, *p* > 0.05, bootstrap portion of samples that have significant correlation was 23.2%, the mean of significant correlation value is 0.62, *N* = 361 PT cells, 5 mice; enriched animals *r* = −0.37, *p* > 0.05 bootstrap portion of samples that have significant correlation was 24.3%, the mean of significant correlation value is −0.75, *N* = 400 PT cells, 6 mice; Fig. 3*F*). Finally, to quantify the information available in population activity, we used Fisher LDA to predict the animal’s decision from either AT or PT cell population activity on a single-trial basis (see Materials and Methods). Consistent with single-cell encoding results, in enriched animals only, an ideal observer can predict the animal’s decision with higher accuracy using AT cell population than using PT cells (control PT bootstrap mean 56%, error bar: 2.5th percentile 35%, and 97.5th percentile 71%, AT bootstrap mean 56%, error bar: 2.5th percentile 36% and 97.5th percentile 68%, *N* = 60 cells, 5 mice; enriched PT bootstrap mean 67%, error bar: 2.5th percentile 62%, and 97.5th percentile 74%, AT bootstrap mean 72%, error bar: 2.5th percentile 66% and 97.5th percentile 78%, *N* = 108 cells, 6 mice; Fig. 3*H*). These results suggest that EE may improve the animal’s sensory acuity by increasing the decision-information capacity in AT cells.

### Tactile environment enrichment enhances decision coding in the premotor cortex

**Figure 4.**
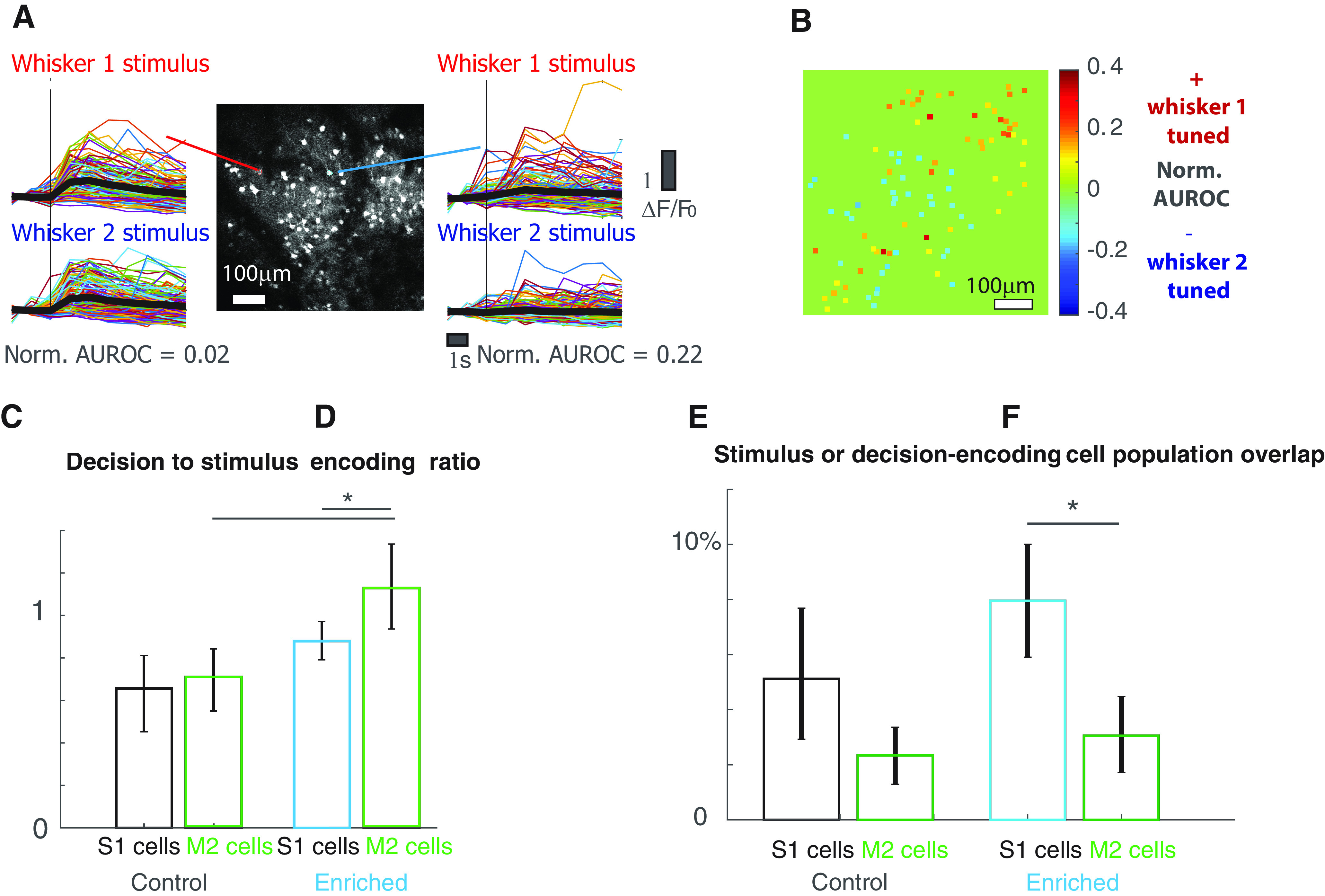
Premotor cells encode decision. ***A***, An example two-photon calcium image of vM2 L2/3 cells in an awake, head-fixed mouse. Some cells respond vigorously to a single whisker stimulation (left), but exhibits little whisker tuning, while others exhibit some moderate extent of whisker preference (right). Thin colored lines are single trials of calcium trace, thick black lines are trial-averaged. ***B***, An example of L2/3 cell whisker tuning in vM2. Unlike vS1, cells tuned to a given whisker do not exhibit any topographical organization. ***C***, In control animals, vM2 cells on average do not encode the animal’s decision relative to stimulus information more than vS1 cells do (control vS1 cells bootstrap mean 0.66, error bar: 2.5th percentile 0.46, and 97.5th percentile 0.81, *N* = 2826 cells; 5 mice; vM2 cells bootstrap mean 0.71, error bar: 2.5th percentile 0.55 and 97.5th percentile 0.84, *N* = 1842 cells, 4 mice). ***D***, In enriched animals, vM2 cells on average encode the animal’s decision more than vS1 cells do (enriched vS1 cells: bootstrap mean 0.88, error bar: 2.5th percentile 0.79, and 97.5th percentile 0.97, *N* = 4266 cells, 6 mice; vM2 cells bootstrap mean 1.13, error bar: 2.5th percentile 0.94 and 97.5th percentile 1.34, *N* = 3972 cells, 7 mice, *p* < 0.05). ***E***, In control animals, the overlap between cellular populations encoding stimulus and decision is not different between vM2 and vS1 (control vS1 cells bootstrap mean 5.1%, error bar: 2.5th percentile 2.9%, and 97.5th percentile 7.7%, *N* = 2826 cells; 5 mice; vM2 cells bootstrap mean 2.3%, error bar: 2.5th percentile 1.3% and 97.5th percentile 3.3%, *N* = 1842 cells, 4 mice, *p* > 0.05). ***F***, In enriched animals, the cellular population encoding stimulus and decision become more separated in vM2 than in vS1, quantified as % of overlap between stimulus-encoding cells and decision-encoding cells (enriched vS1 cells: bootstrap mean 8.0%, error bar: 2.5th percentile 5.9%, and 97.5th percentile 10%, *N* = 4266 cells, 6 mice; vM2 cells bootstrap mean 3.1%, error bar: 2.5th percentile 1.7% and 97.5th percentile 4.5%, *N* = 3972 cells, 7 mice, *p* < 0.05). *p < 0.05.

Because vS1 cells primarily encode the stimulus feature rather than the animal’s decision, we investigated the premotor cortex. It forms reciprocal projections with S1 (Reep et al., 1984, 1987, 1990) and performs critical functions in decision-making (Sul et al., 2011; Murakami et al., 2014) and sensory perception (Vargo et al., 1988; Manita et al., 2015). In a separate group of task-performing animals, we imaged single cell activity in vM2 using two-photon calcium imaging. Figure 4*A* shows an example vM2 imaging session, where cells respond to a single whisker stimulation. Some cells exhibit whisker tuning preference (Fig. 4*A*, right), while others do not (Fig. 4*A*, left). Unlike vS1, vM2 does not have a spatial topography for individual whisker stimulus, as shown in an example tuning map in Figure 4*B*.

**Figure 5.**
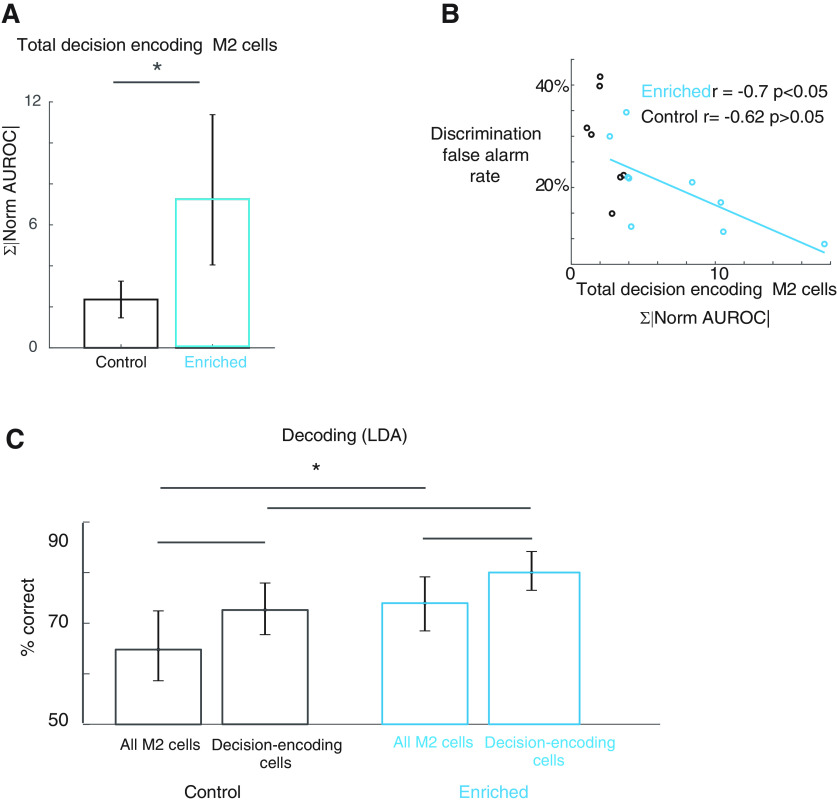
EE increases decision coding in vM2 cells. ***A***, The total amount of information encoded about the animal’s decision in vM2 cells is greater in enriched animals (control bootstrap mean 2.36, error bar: 2.5th percentile 1.46 and 97.5th percentile 3.25, *N* = 1842 cells, 4 mice; enriched bootstrap mean 7.25, error bar: 2.5th percentile 4.04 and 97.5th percentile 11.37, *N* = 3972 cells, 7 mice, *p* < 0.05). ***B***, In enriched animals, the total amount of information encoded about the animal’s decision in vM2 cells predict the animal’s false alarm rate in discrimination task (control *r* = −0.62, *p* > 0.05, in multilevel hierarchical bootstrapped samples, portion of samples that have significant correlation was 25%, the mean of significant correlation value is −0.7, *N* = 1842 cells, 4 mice; enriched *r* = −0.7, *p* < 0.05, in multilevel hierarchical bootstrapped samples, portion of samples that have significant correlation was 54%, the mean of significant correlation value is −0.81, *N* = 3972 cells, 7 mice). ***C***, Using either randomly sampled vM2 cells or decision-encoding cells only, Fisher LDA is used to classify false-alarm and correct rejection trials. For any given session, the number of each type of cells are kept the same. Using either cell type, the decoder predicts a single trial more accurately in enriched animals (control: random cells bootstrap mean 65%, error bar: 2.5th percentile 59%, and 97.5th percentile 73%, decision cells bootstrap mean 73%, error bar: 2.5th percentile 68% and 97.5th percentile 78%, *N* = 131 cells, 4 mice; enriched random cells bootstrap mean 74%, error bar: 2.5th percentile 68%, and 97.5th percentile 80%, decision cells bootstrap mean 80%, error bar: 2.5th percentile 77% and 97.5th percentile 84%, *N* = 510 cells, 7 mice). In general, the decoder performs better using decision cells than using randomly sampled cells. *p < 0.05.

In enriched animals, vM2 cells primarily encode decision rather than stimulus feature, and encode more decision than vS1 cells (decision to stimulus encoding ratio, enriched vS1 cells: bootstrap mean 0.88, error bar: 2.5th percentile 0.79, and 97.5th percentile 0.97, *N* = 4266 cells, 6 mice; vM2 cells bootstrap mean 1.13, error bar: 2.5th percentile 0.94 and 97.5th percentile 1.34, *N* = 3972 cells, 7 mice, *p* < 0.05; Fig. 4*D*). On average, cells in vM2 of enriched animals also encode more decision information than their control counterparts (control vS1 cells bootstrap mean 0.66, error bar: 2.5th percentile 0.46, and 97.5th percentile 0.81, *N* = 2826 cells; 5 mice; vM2 cells bootstrap mean 0.71, error bar: 2.5th percentile 0.55 and 97.5th percentile 0.84, *N* = 1842 cells, four mice; Fig. 4*C*). Furthermore, compared with vS1 cells, vM2 cells have a smaller overlap between populations that encode stimulus and those that encode decision (% overlap enriched vS1 cells: bootstrap mean 8.0%, error bar: 2.5th percentile 5.9%, and 97.5th percentile 10%, *N* = 4266 cells, 6 mice; vM2 cells bootstrap mean 3.1%, error bar: 2.5th percentile 1.7% and 97.5th percentile 4.5%, *N* = 3972 cells, 7 mice, *p* < 0.05; Fig. 4*F*). These results suggest that vM2 cells, downstream to vS1, may become more functionally specialized in the type of information they encode. Again, these patterns do not hold for control animals (control vS1 cells bootstrap mean 5.1%, error bar: 2.5th percentile 2.9%, and 97.5th percentile 7.7%, *N* = 2826 cells; 5 mice; vM2 cells bootstrap mean 2.3%, error bar: 2.5th percentile 1.3% and 97.5th percentile 3.3%, *N* = 1842 cells, 4 mice, *p* > 0.05; Fig. 4*E*). The total amount of decision information encoded in enriched vM2 cells was higher than that in control animals (control bootstrap mean 2.36, error bar: 2.5th percentile 1.46 and 97.5th percentile 3.25, *N* = 1842 cells, 4 mice; enriched bootstrap mean 7.25, error bar: 2.5th percentile 4.04 and 97.5th percentile 11.37, *N* = 3972 cells, 7 mice, *p* < 0.05; Fig. 5*A*), and correlated with the animal’s behavior (control *r* = −0.62, *p* > 0.05, in multilevel hierarchical bootstrapped samples, portion of samples that have significant correlation was 25%, the mean of significant correlation value is −0.7, *N* = 1842 cells, four mice; enriched *r* = −0.7, *p* < 0.05, in multilevel hierarchical bootstrapped samples, portion of samples that have significant correlation was 54%, the mean of significant correlation value is −0.81, *N* = 3972 cells, 7 mice; Fig. 5*B*). We analyzed population activity in vM2 to predict animal decision on single-trial basis. The LDA decoder performs better using vM2 cells in enriched animals than those in control animals. The decoder also shows higher accuracy when using decision-encoding cells than using the same number of randomly selected vM2 cells (control: random cells bootstrap mean 65%, error bar: 2.5th percentile 59%, and 97.5th percentile 73%, decision cells bootstrap mean 73%, error bar: 2.5th percentile 68% and 97.5th percentile 78%, *N* = 131 cells, four mice; enriched random cells bootstrap mean 74%, error bar: 2.5th percentile 68%, and 97.5th percentile 80%, decision cells bootstrap mean 80%, error bar: 2.5th percentile 77% and 97.5th percentile 84%, *N* = 510 cells, 7 mice; Fig. 5*C*). These results suggest that EE enhances the ability of vM2 cells to encode the animal’s decision. During enrichment, vM2 cells develop more specialized cellular populations dedicated to stimulus encoding and decision-making respectively. These mechanisms may improve sensory acuity in behaving animals.

## Discussion

EE is known to be beneficial to sensory functions (Mandairon et al., 2006a,b; Sale et al., 2007; Cai et al., 2009; Baroncelli et al., 2012; Tognini et al., 2012). Therefore, a full understanding of the neural processes underlying improved sensory acuity induced by enrichment is critical. We aim to contribute to this question by demonstrating improvements of tactile sensory stimuli discrimination and its underlying information coding at single-cell level in both vS1 and vM2.

In many sensory modalities, such as audition (Cai et al., 2009) and olfaction (Mandairon et al., 2006a,b), EE improves stimulus discrimination in animal behavior. However, tactile enrichment seemed not to improve texture discrimination in rodents (Finger and Fox, 1971; Bourgeon et al., 2004). Although texture discrimination is an ethologically natural task, it is possibly not difficult enough for the animal to reflect the full effect of enrichment. We designed a single-whisker spatial discrimination task that was difficult enough for the normal-housed animal, so that EE significantly improved the task performance. Strictly speaking, the laboratory housing reflects a form of sensory deprivation, whereas our enriched housing rather imitates the “normal” tactile environment.

To uncover the underlying neural processes of how EE improved tactile discrimination, we analyzed stimulus-information and decision-information coding at the single cell level. Cells that are not tuned to their principal whisker are of particular interest. The cortical column is one of the most prominent organizations in the neocortex and well researched. In contrast, relatively little is known about the horizontal flow of sensory information between columns. In our model of barrel cortex, this is reflected in L2/3 cells that are tuned to an adjacent, or even multiple whiskers. Unlike L4, L2/3 cells form a salt-and-pepper-like tuning map (Sato et al., 2007; Kerr et al., 2007; Clancy et al., 2015). Because of the nature of precise spatial somatotopy in L4 of the barrel system, it may be intuitive to suppose this salt-and-pepper-like non-principal whisker tuning may obscure spatial acuity in sensation. Thus, whether spatially-heterogeneous tuning is simply a by-product of organizational imprecision, or it serves a purpose in sensory processing remained as a question. Our study addresses this question. We show that stimulus-evoked activity in adjacent cortical columns does not impede the spatial acuity in the animal’s sensation. On the contrary, adjacent-whisker-tuned cells encode important neural correlates of improved sensory acuity. Importantly, EE enhanced decision-coding in adjacent-whisker-tuned cells, but not in their PT counterparts.

Because EE is known to improve cognitive behavior (Van Praag, 2000; Leggio et al., 2005), it is possible that the improvement in adjacent-whisker discrimination was because of general cognitive improvements, such as better memory or impulse control. As a control, we tested the animals in a whisker detection task, where they were trained to withhold licking when no whisker stimulus was presented. We found that the detection false alarm rates were similarly high in enriched animals compared with ones in control animals (Fig. 1*F*). Thus, it is not likely that the reduced false alarm rate during the discrimination task was mainly because of a better impulse control, but such possibilities should not be discarded.

Although other studies have also addressed how EE may affect multiwhisker tuning or a single whisker receptive field, the results are divided in the literature. Some showed enriched (LeMessurier et al., 2019) or natural housing (Polley et al., 2004) spatially sharpened whisker-evoked activity in vS1, and speculated that a sharpened spatial representation may improve tactile sensing. Others showed EE broadened a single whisker spatial span (Guic et al., 2008; Devonshire et al., 2010). It is worth noting that these studies lacked behavior tests or neural recordings in awake, behaving animals. In addition, these results were recorded under different anesthesia. Some even involved whisker trimming, which might cause massive cortical reorganization (Diamond et al., 1993). Any of these factors can cause major discrepancies in the conclusions about cellular plasticity in vS1. Our study shows that enrichment increases adjacent-whisker-tuning in vS1 cells in awake mice performing a spatial acuity task.

What are some possible neural mechanisms by which adjacent-whisker-tuned cells improve sensory acuity during enrichment? Previously it has been shown that in short-term sensory adaptation, less spatially overlapped whisker representation in vS1 sharpens the sensory acuity in behaving rats (Ollerenshaw et al., 2014). However, the neural mechanisms in adaptation involve short-term thalamocortical synaptic depression and brain state modulation, which are not likely to be contributing factors to present results. Furthermore, the present study imaged single-cell activity in behaving animals whereas the previous study relied on wide-field voltage sensitive dye imaging in anesthetized animals separate from the behaving group.

AT or multiwhisker-tuned cells have been speculated to integrate information across columns. It has been suggested that whisker-evoked activity in adjacent columns may provide lateral inhibition when processing multiwhisker input (Brumberg et al., 1996; Moore and Nelson, 1998; Adesnik and Scanziani, 2010) and help propagate and maintain recurrent activity (Wester and Contreras, 2012). Computational studies have speculated that multiwhisker-tuned cells sharpen tuning in primary visual cortical cells (Chelaru and Dragoi, 2008), and cross-column activity may enable object recognition (Hawkins et al., 2017).

In enriched animals, the potentiated response in adjacent columns likely arises, at least partially, from long-term potentiation of cross-column synapses in L2/3. This may help adjacent-whisker-tuned cells to integrate cross-column information and compare the inputs from multiple columns. The sources of input to AT L2/3 cells most likely include excitatory inputs from L2/3 cells in adjacent columns, because L2/3 cells span their axons extensively into neighboring columns (Petersen, 2007). Other sources of inputs to L2/3 should also be considered. For instance, L4 spiny neurons send projections into adjacent columns in L2/3 (Egger et al., 2008). Subcortical regions, such as the posterior medial (POm) nucleus of the thalamus, have also been shown to provide potent inputs to S1 L2/3 cells (Lu and Lin, 1993; Rubio-Garrido et al., 2009; Wimmer et al., 2010; Sherman and Guillery, 2011; Ohno et al., 2012; Jouhanneau et al., 2014; Zhang and Bruno, 2019). Other important sources of input to L2/3 cells in S1 are other cortical areas, such as primary motor cortex (Veinante and Deschênes, 2003; Petreanu et al., 2009; Lee et al., 2013; Kinnischtzke et al., 2014), secondary somatosensory cortex (Kwon et al., 2016; Yang et al., 2016), and premotor cortex (Manita et al., 2015; discussed below), all of which have been shown to play important roles in the animal’s sensory choice.

Furthermore, L2/3 cells project to layer 5 (L5), and both layers project to other cortical areas (Feldmeyer and Sakmann, 2000; Douglas and Martin, 2004; Manita et al., 2015). An important function of these adjacent-whisker-tuned cells may be to amplify sensory information across the entire layer, thus enhancing outputs to both L5 and to other cortical areas, and maintain reverberant feedback loop activity between vS1 and other cortical areas.

Of all the downstream targets of vS1, vM2 is of particular interest. It is involved in the neural process underlying perceptual choice (Manita et al., 2015), which is an important intermediate step between stimulus representation in the cortex and the animal’s sensation. The feedback activity from higher-order cortices such as M2 to primary sensory area is known to be critical for sensory processing (Pascual-Leone and Walsh, 2001; Gilbert and Sigman, 2007; Dehaene and Changeux, 2011; Zagha et al., 2013; Manita et al., 2015; Kwon et al., 2016). Interestingly, in vM2 of enriched animals, not only does the amount of decision information increase, there is also a bifurcation of cellular populations encoding stimulus and decision respectively. This functional specialization could contribute to a more dedicated decision-making process, and possibly enhance the feedback of decision information to vS1. Because adjacent-whisker-tuned cells in vS1 carry more decision information than principal-whisker-tuned cells, they may be the recipient of feedback information from vM2. The increased response in adjacent columns may indicate that AT cells could form a horizontal network that spreads the feedback information across the supragranular layers of vS1. The enhancement of decision information coding in both adjacent-whisker-tuned cells in vS1 and vM2 cells suggests that a possible mechanism of EE is by enhancing the connection between vS1 and vM2.

In summary, our study advances the understanding of neural mechanism of EE in sensory functions. We show that in awake, behaving mice, tactile enrichment improves tactile stimulus feature discrimination.

## Synthesis

Reviewing Editor: Mary Heinricher, Oregon Health & Science University Decisions are customarily a result of the Reviewing Editor and the peer reviewers coming together and discussing their recommendations until a consensus is reached. When revisions are invited, a fact-based synthesis statement explaining their decision and outlining what is needed to prepare a revision will be listed below. The following reviewer(s) agreed to reveal their identity: NONE.

In this revised manuscript, the authors harness the somatotopic organization of the mouse whisker-barrel system to evaluate the hypothesis that “environmental enrichment evokes functional plasticity by enhancing input of adjacent columns in L2/3 cells.” The authors first injected a GCaMP6-encoding virus into the superficial layers of either the barrel (SI) or the premotor cortex (vM2) and then exposed the mice either to standard housing or to an enriched environment for 40 hrs/week for 4 weeks. The water-deprived mice were then trained to perform a “go/no-go” licking task in response to stimulation of two adjacent whiskers while activity of LII/III neurons in SI or in the premotor cortex was imaged through a cranial window. The authors find that in mice exposed to an enriched environment, the probability of “false alarm” - licking in response to stimulation of the adjacent, as opposed to the anatomically corresponding principal, whisker - decreases. In addition, adjacent whisker representation in LII/III neurons increases after enrichment. In vM2, LII/III neurons in enriched mice carry more information about the behavioral decision than those in control mice. Together, these data suggest a significant impact of environmental enrichment on processing of sensory information in the superficial layers of SI and on the link between information transfer and behavior via vM2.

The statistical analyses have been improved through the use of bootstrapping techniques to account for data dependence.

The Discussion section still needs to address some alternative interpretations. First, the decreased “false positive” rate may reflect improved impulse control in mice exposed to enrichment. Second, the control mice are essentially exposed to sensory deprivation (individual housing and empty cages). As with many environmental enrichment studies, the effect of enrichment may actually represent the normal state of the system, and the control groups represent the effect of sensory deprivation. Finally, the authors do not really provide evidence that increased responsiveness to adjacent whisker deflections arises from the adjacent cortical column, as they propose in their hypothesis. It is possible that it arises from selective modulation of synapses arising from LIV barrel neurons or from sources outside SI. Each of these three issues should be addressed in the manuscript.

It remains unclear whether mice were used in both behavioral and imaging groups. Please clarify.

A photograph of an “enriched” cage is now provided, but the lack of water, bedding and food raises questions as to whether this is entirely representative.

A graphical representation of the difference in tuning should be included, which would allow the readers to judge whether this is a significant and meaningful difference.

Additional comments related to organization and presentation of the manuscript.

As noted in the prior review, the final paragraph of the Introduction should not be a summary of findings. Please finish this section with the aim of the study and any predictions. Per journal guidelines: “The Introduction should briefly indicate the objectives of the study and provide enough background information to clarify why the study was undertaken and what hypotheses were tested.”

The data in bar graphs may be better represented through the use of box-and-whisker (no pun intended) plots.

Finally, the manuscript remains very difficult to read, with an abundance of run-on sentences and grammatical mistakes. Below is a sample just from the first few lines. A professional English editor might be helpful in making the manuscript more accessible.

Lines 4-9: run on sentence

Lines 14-17: What does “simultaneously” refer to in this sentence?

Lines 35-37: this sentence is not grammatically correct

Lines 51-52: “..., especially LII/III, exhibits...”

Lines 56-60: this sentence is not grammatically correct

Lines 61-72: this sentence is both run-on and not grammatically correct

One of the headings in Results still refers to “homogeneous” cells
